# Effects of Microplate Type and Broth Additives on Microdilution MIC Susceptibility Assays

**DOI:** 10.1128/AAC.01760-18

**Published:** 2018-12-21

**Authors:** Angela Kavanagh, Soumya Ramu, Yujing Gong, Matthew A. Cooper, Mark A. T. Blaskovich

**Affiliations:** a Institute for Molecular Bioscience, The University of Queensland, St. Lucia, Queensland, Australia

**Keywords:** MIC, additive effects, broth microdilution assay, microtiter plate effects

## Abstract

The determination of antibiotic potency against bacterial strains by assessment of their minimum inhibitory concentration normally uses a standardized broth microdilution assay procedure developed more than 50 years ago. However, certain antibiotics require modified assay conditions in order to observe optimal activity. For example, daptomycin requires medium supplemented with Ca^2+^, and the lipoglycopeptides dalbavancin and oritavancin require Tween 80 to be added to the growth medium to prevent the depletion of free drug via adsorption to the plastic microplate. In this report, we examine systematically the effects of several different plate types on microdilution broth MIC values for a set of antibiotics against Gram-positive and Gram-negative bacteria, both in medium alone and in medium supplemented with the commonly used additives Tween 80, lysed horse blood, and 50% human serum. We observed very significant differences in measured MICs (up to 100-fold) for some lipophilic antibiotics, such as the Gram-positive lipoglycopeptide dalbavancin and the Gram-negative lipopeptide polymyxins, and found that nonspecific binding plates can replace the need for surfactant additives. Microtiter plate types and any additives should be specified when reporting broth dilution MIC values, as results can vary dramatically for some classes of antibiotics.

## Abstract (precis)

The determination of antibiotic potency against bacterial strains by assessment of their minimum inhibitory concentration normally uses a standardized broth microdilution assay procedure developed more than 50 years ago. However, certain antibiotics require modified assay conditions in order to observe optimal activity.

## INTRODUCTION

Antibiotic effectiveness against bacterial strains is routinely assessed by a variety of methods, including disk diffusion assays, gradient diffusion methods (e.g., the commercial bioMérieux Etest or Thermo Scientific Oxoid M.I.C.Evaluator strip systems) and automated susceptibility testing (1) (such as the bioMérieux Vitek, BD Phoenix, Beckman Coulter MicroScan, and Thermo Fisher Scientific Sensititre systems). The gold standard reference assays are considered to be agar and broth dilution assays, in which the antibiotic of interest is serially diluted in 2-fold steps in agar or broth, with each plate or tube/well then inoculated with a defined number of bacteria. The MIC is then measured as the lowest concentration of drug that will inhibit visible growth of the organism after overnight incubation. A number of standardized procedures have been published. The most widely referenced is the Clinical and Laboratory Standards Institute (CLSI) M07-A11 (2) and accompanying M100-S28 supplement (3), which include procedures for agar and broth dilution assays. In 2006, the British Society for Antimicrobial Chemotherapy (BSAC) published a document available on their website (4) that also describes agar and broth dilution determinations, an updated version of a 2001 reference (5). Both the BSAC and the European Committee on Antimicrobial Susceptibility Testing (EUCAST) have methods available on their websites for standardized disk diffusion assays. The EUCAST website refers to recommendations from the International Organization for Standardization (ISO) for broth MIC determination for nonfastidious organisms, with modified medium for fastidious organisms (http://www.eucast.org/ast_of_bacteria/mic_determination/?no_cache=1). The relevant ISO guidance document is ISO 20776-1 (6).

The published reference procedures for broth dilution assays do not generally specify the type and nature of the container in which the assays should be conducted, with the CLSI M07-A11 document listing “sterile 13 × 100-mm test tubes” for the macrodilution procedure and “plastic microdilution trays that have round or conical bottom wells” for the microdilution procedure (2). The one exception is a CLSI-EUCAST working group recommendation in 2016 that surfactants should not be included in the reference broth microdilution method for colistin, and that untreated polystyrene (PS) trays should be employed (7). Agar and broth dilution methods are also reported in a 2008 *Nature Protocols* article (8), which is one of the very few published protocols that mentions the potential for plate composition effects on MIC potency. This report showed that cationic antimicrobial peptides (AMPs) have reduced MICs in tissue culture-treated PS plates compared to those in polypropylene (PP) plates, and that the addition of acetic acid/bovine serum albumin (BSA) alleviated this effect (8). The adherence of cationic AMPs to PS (particularly tissue-culture treated PS) is also mentioned in a note in a 2007 *Methods in Molecular Biology* chapter, which recommended the use of PP plates (9).

**FIG 1:**
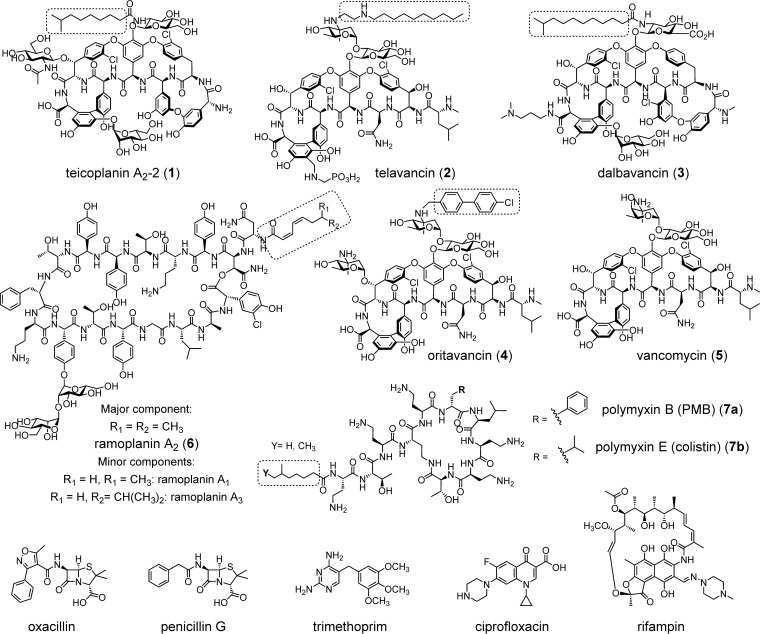
Chemical structures of lipophilic antibiotics and nonlipophilic comparators: teicoplanin (compound 1), telavancin (compound 2), dalbavancin (compound 3), oritavancin (compound 4), vancomycin (compound 5), ramoplanin (compound 6), polymyxin B (compound 7a), colistin (compound 7b), oxacillin, penicillin G, trimethoprim, ciprofloxacin, and rifampin. Lipophilic groups are highlighted with a dashed box.

Some antibiotics, such as the lipoglycopeptides teicoplanin (compound 1), telavancin (compound 2), dalbavancin (compound 3), oritavancin (compound 4), and ramoplanin (compound 6) (Fig. 1), must be solubilized in dimethyl sulfoxide (DMSO) or 0.002% polysorbate 80 (Tween 80) in water to prevent adherence to plastic surfaces, including assay plates (10–14), with other additives, such as 2% lysed horse blood (LHB) (10) or 0.02% bovine serum albumin (BSA) (15, 16) found to have a similar blocking effect as surfactant. Notably, the closely related glycopeptide vancomycin (compound 5), without a lipophilic moiety, does not require surfactant supplement. Similarly, MIC determinations of the lipopeptide polymyxin class of antibiotics (polymyxin B [compound 7a] and polymyxin E or colistin, compound 7b) have been found to be affected by additives (0.002% polysorbate) (17–20) and assay container composition (21). During the preparation of this paper, two new reports described container effects on polymyxin MIC determinations (22, 23).

**FIG 2:**
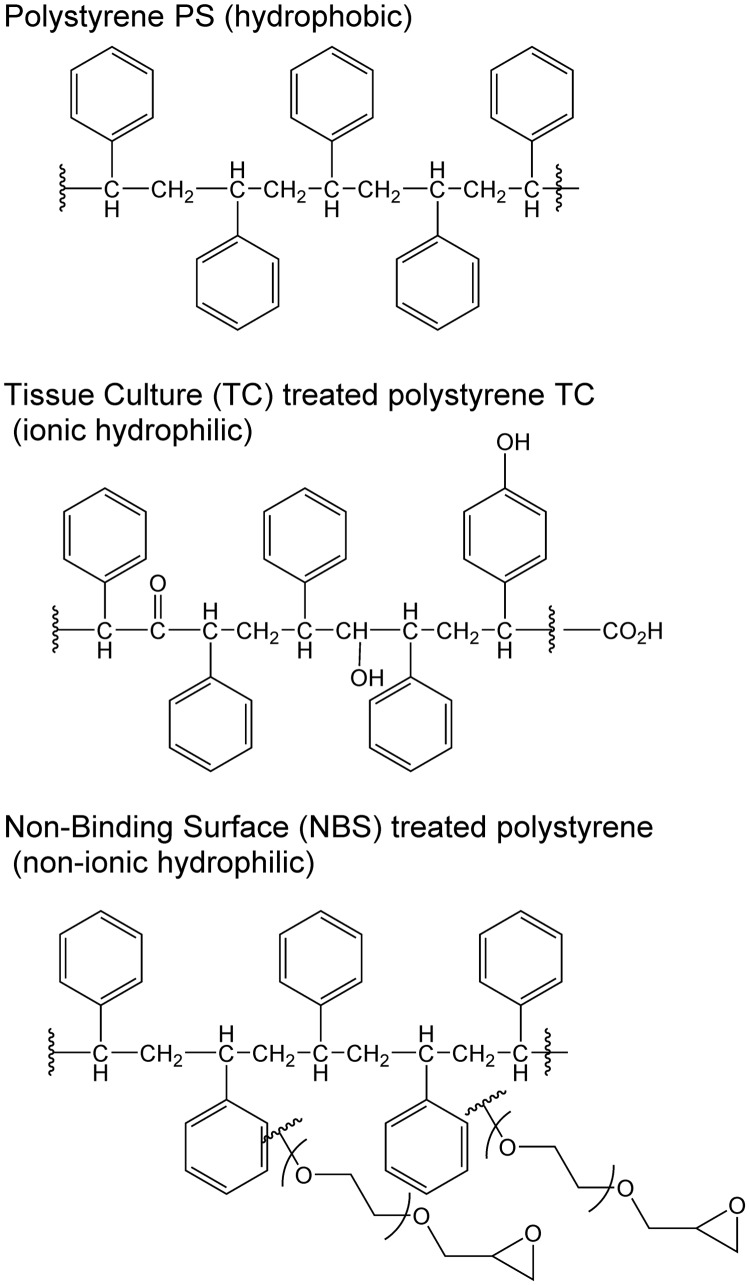
Plate surface modifications of polystyrene for tissue culture (76) and nonbinding surface (77) plates.

Microtiter plates used in broth microdilution assays are generally made from PS, but several different types of surface modifications are available. For example, Corning offers over 10 types of surface treatments for microplates, many of them designed to specifically bind cells or biomolecules. Untreated PS is considered a medium binding surface that is hydrophobic and binds biomolecules through passive interactions. The standard tissue culture-treated (TC-treated) surface, used for the attachment and growth of anchorage-dependent cells, is created by applying a corona discharge that grafts oxygen atoms onto the surface PS chains (Fig. 2) so that the surface becomes hydrophilic and negatively charged. Other binding surfaces include a high binding surface to bind biomolecules that possess ionic groups and/or hydrophobic regions, and surfaces coated with poly-d-lysine, sulfhydryl, carbohydrate, amine, or photoreactive groups that can be used to covalently immobilize biomolecules.

Some surfaces are designed to minimize binding, including Corning’s nonbinding surface (NBS), a proprietary treatment technology that creates a polyethylene oxide-like nonionic hydrophilic surface to minimize nonspecific molecular interactions (Fig. 2). The NBS surface has been compared to untreated PS and PP for the binding of radiolabeled proteins; BSA bound to PS at 450 ng/cm^2^ and to PP at 440 ng/cm^2^ but to the NBS-coated PS at <2.5 ng/cm^2^ (78), so the NBS plate is recommended to reduce protein binding during assays.

Similarly, Thermo Scientific Nunc offers a range of treated plates, generally based on PS, with various degrees of absorption characteristics; the Nunc MiniSorp and GeNunc module surfaces have very low nonspecific binding characteristics, due to specially formulated polyethylene resin. Unfortunately, some plates have optical characteristics unsuitable for MIC determinations requiring optical density or visual readouts of turbidity.

**FIG 3:**
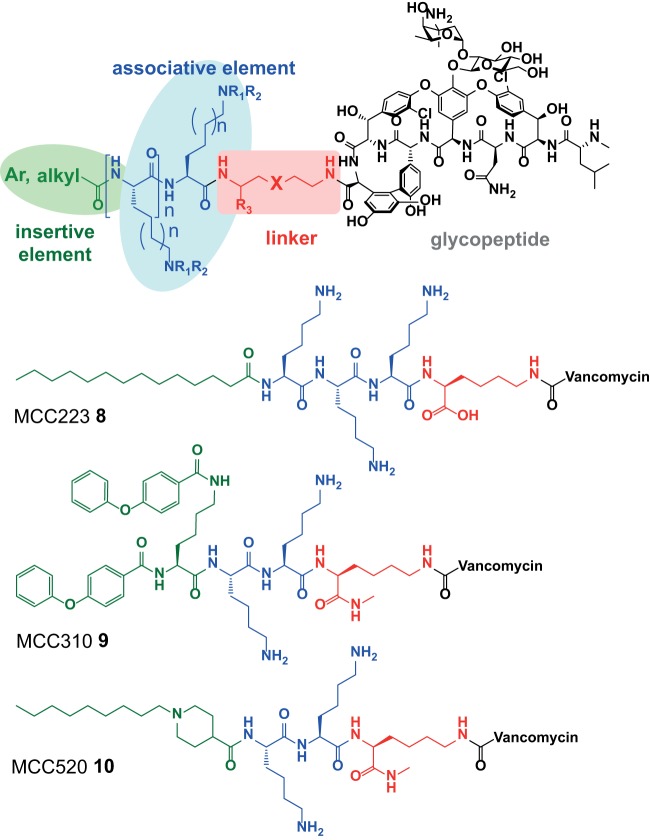
General design of the vancapticins, and specific structures of MCC223, MCC310, and MCC520.

In the course of developing third-generation semisynthetic lipoglycopeptide antibiotics designed to selectively target bacterial membranes (24), our laboratory noted that nonbinding surface (NBS) plates provided significantly improved microdilution MIC values compared to other types of plates, and we also observed significant variations caused by added protein or surfactant depending on the plate type. As a result, we initiated a systematic comparison of several different plate types for microdilution assays, comparing various antibiotics against both Gram-positive and Gram-negative strains, and with/without a number of common additives. We were particularly interested in determining if a common plate type could prevent the need for specialized assay conditions for individual lipophilic antibiotics, driven by our internal drug discovery program on synthetic lipoglycopeptide vancomycin derivatives. These “vancapticins” increase the selectivity of vancomycin toward bacterial membranes by using an attached cationic “associative” peptide sequence terminated with an “insertive” lipophilic group (Fig. 3) (24). They possess high protein binding and a propensity to adhere to plastic surfaces, similar to the second-generation lipoglycopeptides telavancin (compound 2), dalbavancin (compound 3), and oritavancin (compound 4). The ability to avoid additives would simplify assay preparation, preventing errors due to incorrect concentrations of polysorbate. It would also avoid potential unexpected effects of added surfactants, given that a nonionic polyethylene glycol surfactant (Triton X-100) similar to Tween 80 (T80) used to avoid compound aggregation during other types of screening assays has been shown to unpredictably affect assay results (25). More importantly, it would enable better standardization of conditions and comparison of antimicrobial activity profiles between laboratories if a single plate type, with no need for broth additives, could be adopted.

## RESULTS

Our studies were designed to examine the effects of different plate types on broth microdilution MIC determinations. Initially, we selected seven different PS-based plate types, as follows: Corning untreated flat-bottom, Corning TC-treated flat-bottom, Corning NBS-treated flat-bottom, Nunc untreated flat-bottom, Nunc TC-treated U-bottom, Nunc TC-treated flat-bottom, and Trek Diagnostics untreated flat-bottom plates. For each plate type, one Gram-positive organism was tested against seven antibiotics and one Gram-negative organism against seven antibiotics (see Fig. S1 in the supplemental material for experimental design). Antibiotics were selected to include those previously reported to exhibit plate- or surfactant-based MIC variations, as well as examples expected to not show an effect. All assays were conducted in both Mueller-Hinton broth (MHB) and in MHB supplemented with 0.002% T80 to see if the plate type could obviate surfactant. The second set of assays (Fig. S1) examined whether differences between plates seen in the first experiments were consistent across bacterial strains. Three plate types from one manufacturer (Corning untreated, TC treated, and NBS treated) were compared using the same sets of antibiotics but with six additional Gram-positive and three additional Gram-negative strains, again using MHB medium with and without T80. The final experiments (Fig. S1) compared the effects of different additive used to assess the effectiveness of antibiotics under physiological conditions, 50% human serum or 2% lung surfactant. These were done in four plate types (Trek PS untreated or Corning PS untreated, TC treated, and NBS treated), and were compared in MHB, MHB with added 0.002% T80, and MHB with added 2% LHB.

### Experiment 1, initial plate comparison with or without Tween 80.

**FIG 4:**
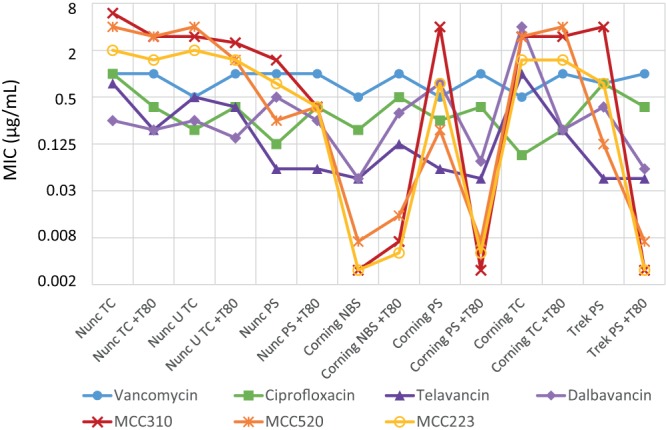
Comparison of antibiotic MICs versus MRSA (ATCC 43300) determined in seven different plate types, with and without the addition of 0.002% Tween 80.

The first set of experiments compared MIC values determined in seven different plate types against one Gram-positive (methicillin-resistant Staphylococcus aureus [MRSA] ATCC 43300) and one Gram-negative (Escherichia coli ATCC 25922) organism conducted in MHB with and without 0.002% T80, using two sets of the following seven antibiotics for the two different classes of bacteria: vancomycin, ciprofloxacin, telavancin, dalbavancin, MCC233, MCC310, and MCC520 for MRSA; and colistin, ciprofloxacin, oxacillin, trimethoprim, polymyxin B, penicillin, and rifampin for E. coli (note that oxacillin and penicillin G generally have poor Gram-negative activity; they were included to assess if surfactant additives or plate types had synergistic effects, via damage to the bacterial membranes, that increased their potency by providing greater access to the periplasm/peptidoglycan). Literature value ranges for these antibiotics against MRSA and E. coli are listed in Table 1, with plate results tabulated in Table 2 and visualized in Fig. 4.

**TABLE 1:** Literature MIC values for tested antibiotics

Antibiotic	MIC (µg/ml) (reference[s])	
	MRSA	E. coli
Vancomycin	2 (62)	
Ciprofloxacin	0.5 (63)	0.03–0.06 (64, 65)
Telavancin	0.06 (66, 67)	
Dalbavancin	0.06 (68, 69)	
Colistin		0.5–2 (70)
Oxacillin		>128 (71)
Trimethoprim		0.06–0.25 (72)
Polymyxin B		0.25–1 (73)
Penicillin G		16–64 (71)
Rifampin	0.015 (62)	2 (74, 75)

**TABLE 2:** Comparison of 7 plate types regarding MIC versus MRSA ATCC 43300 in Mueller-Hinton broth in the presence and absence of Tween 80 (polysorbate 80)

Plate	Medium	MIC (µg/ml) by antibiotic						
		Vancomycin	Ciprofloxacin	Telavancin	Dalbavancin	MCC223	MCC310	MCC520
Nunc flat TC	−T80	1–2	1	0.5–1	0.25	2	4–8	4
	+T80	1	0.25–0.5	0.125– 0.25	0.125–0.25	1–2	2–4	2–4
Nunc U TC	−T80	0.5	0.125–0.25	0.5	0.25	2	2–4	4
	+T80	1	0.25–0.5	0.25–0.5	0.06–0.25	1–2	1–4	1–2
Nunc flat PS	−T80	1	0.125	0.06	0.5	0.5–1	1–2	0.25
	+T80	1	0.25–0.5	0.06	0.25	0.25–0.5	0.25–0.5	0.25–0.5
Corning flat NBS	−T80	0.5	0.125–0.25	0.03–0.06	0.03–0.06	≤0.003	≤0.003	0.007
	+T80	1	0.5	0.125	0.125–0.5	0.003–0.007	0.007–0.015	0.015
Corning flat PS	−T80	0.5	0.25	0.06	0.5–1	0.5–1	4	0.125–0.25
	+T80	1–2	0.25–0.5	0.03–0.06	0.03–0.125	0.003–0.007	≤0.003	0.007
Corning flat TC	−T80	0.5	0.06–0.125	1	4	1–2	2–4	2–4
	+T80	1	0.125–0.5	0.125–0.25	0.125–0.25	1–2	2–4	4
Trek flat PS	−T80	0.5–1	0.125–0.5	0.03–0.06	0.25–0.5	0.5–1	4	0.125
	+T80	1	0.25–0.5	0.03–0.06	0.06	≤0.003	≤0.003	0.007–0.015

The control antibiotics with low protein binding and nonlipophilic characteristics, e.g., ciprofloxacin and vancomycin, showed little variations in MIC regardless of plate type or addition of T80 (vancomycin, around 1 µg/ml; ciprofloxacin, around 0.125 µg/ml). In contrast, large plate effects were observed for lipophilic antibiotics against both MRSA and E. coli, with some antibiotics (dalbavancin, the vancapticins, colistin, and polymyxin B) showing much more potent activity in NBS plates. The nontreated plates showed varied effects depending on the manufacturer, while the TC-coated plates uniformly showed decreased antibiotic activity. The presence of Tween 80 had little impact on NBS plate results (although MIC activity was slightly reduced in some cases), nor in Corning or Nunc TC plates, but resulted in much greater potency of some antibiotics in Corning and Trek PS plates, with less effect in Nunc PS plates. Notably, antibiotics that had improved potency with the addition of Tween 80 in untreated PS plates showed equivalent MIC values in NBS plates without added surfactant. Given that the control antibiotics with low protein binding and nonlipophilic characteristics showed little variation, it would appear to be unlikely that the plate or Tween itself is having a synergistic effect on antibacterial activity.

For the assays against MRSA, telavancin in all 3 untreated PS plates showed values around 0.06 µg/ml with and without added T80, similar to levels in NBS plates, while TC plates without additive produced higher values (0.5 to 1 µg/ml), which were reduced when T80 was added (0.125 µg/ml). Dalbavancin and the vancapticins showed much more pronounced variations, with >4-fold improvements (and up to >1,000-fold) when T80 was added to PS plates from two of the three manufacturers; improvements in Nunc PS plates were substantially less striking (1- to 8-fold). The Corning NBS plates without additive gave values comparable to those with PS plus T80 (e.g., ≤0.003 to 0.03 µg/ml). The addition of T80 to NBS plates resulted in 2-fold or greater reductions in potency. The TC plates consistently gave MICs of ≥2 µg/ml for these antibiotics, with minimal improvements upon the addition of T80.

**FIG 5:**
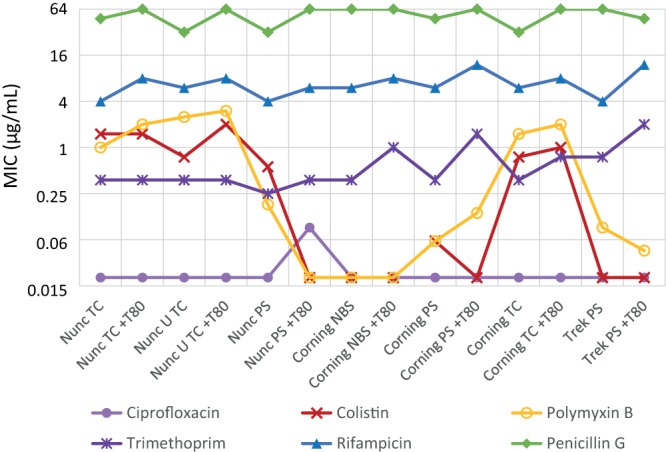
Comparison of antibiotic MICs versus *E. coli* (ATCC 25922) determined in seven different plate types, with and without the addition of 0.002% Tween 80 (note oxacillin not seen as >64 g/ml).

Similar variations were seen in the antibiotics tested against E. coli (Table 3 and Fig. 5). Ciprofloxacin, oxacillin, penicillin G, trimethoprim, and rifampin showed little variation across plate types, with or without added T80. In contrast, colistin (and, to a lesser extent, polymyxin B) showed the same trends as the lipoglycopeptides, as follows: PS plates gave more potent values (≤0.03 to 1 µg/ml) than TC plates (0.5 to 4 µg/ml), and the addition of T80 generally improved activity in PS plates (≤0.03 to 0.25 µg/ml) but not in TC or NBS plates. The NBS plates gave values equivalent to the best PS plus T80 results (≤0.03 µg/ml).

**TABLE 3:** Comparison of 7 plate types regarding MIC versus *E. coli* ATCC 25922 in Mueller-Hinton broth in the presence and absence of Tween 80 (polysorbate 80)

Plate	Medium	MIC (µg/ml) by antibiotic						
		Colistin	Ciprofloxacin	Oxacillin	Trimethoprim	Polymyxin B	Penicillin G	Rifampin
Nunc flat TC	−T80	1–2	≤0.03	>64	0.25–0.5	1	32–64	4
	+T80	1–2	≤0.03	>64	0.25–0.5	2	64	8
Nunc U TC	−T80	0.5–1	≤0.03	>64	0.25–0.5	1–4	32	4–8
	+T80	2	≤0.03	>64	0.25–0.5	2–4	64	8
Nunc PS	−T80	0.125–1	≤0.03	>64	0.25	0.125–0.25	32	4
	+T80	≤0.03	0.06–0.125	>64	0.25–0.5	0.03–0.06	64	4–8
Corning NBS	−T80	≤0.03	≤0.03	>64	0.25–0.5	≤0.03	64	4–8
	+T80	≤0.03	≤0.03	>64	1	≤0.03	64	8
Corning PS	−T80	0.06	≤0.03	>64	0.25–0.5	0.06	32–64	4–8
	+T80	≤0.03	≤0.03	>64	1–2	0.03–0.25	64	8–16
Corning TC	−T80	0.5–1	≤0.03	>64	0.25–0.5	1–2	32	4–8
	+T80	1	≤0.03	>64	0.5–1	2	64	8
Trek PS	−T80	≤0.03	≤0.03	>64	0.5–1	0.06–0.125	64	4
	+T80	≤0.03	≤0.03	>64	2	0.03–0.06	32–64	8–16

### Experiment 2, plate plus Tween 80 comparison versus expanded set of bacteria.

In order to examine whether the plate variations in MIC extended across multiple types of bacteria, three plate types (PS, TC, and NBS) from the same manufacturer were compared, using the same combinations of seven antibiotics against either seven Gram-positive (Table S1 and Fig. S2) or four Gram-negative (Table S2 and Fig. S3) bacteria. The results were very similar to those observed from the first experiments across all strains tested, in that more polar antibiotics were generally unaffected by the plate type/additive, while lipoglycopeptides or lipopeptides were least potent in TC-without T80 plates and most potent in PS with T80, with equivalent activity in NBS without T80.

### Experiment 3, plate plus additives comparison.

In assessing the activity of potential antibiotics, it is important to conduct assays in the presence of biological components that the antibiotics will encounter in the human body, namely, human serum (inactivation by protein binding) and lung surfactant (encountered when treating pneumonia). We therefore assessed four plate types (PS, TC, and NBS from Corning, and PS from Trek) in MHB, MHB plus 50% human serum (HS), and MHB plus 2% artificial lung surfactant (LS). For this experiment, in addition to testing the activity with no additive, and with T80, we also tested in the presence of 2% lysed horse blood (LHB), which was previously reported to have the same blocking effect as surfactant (10).

**FIG 6:**
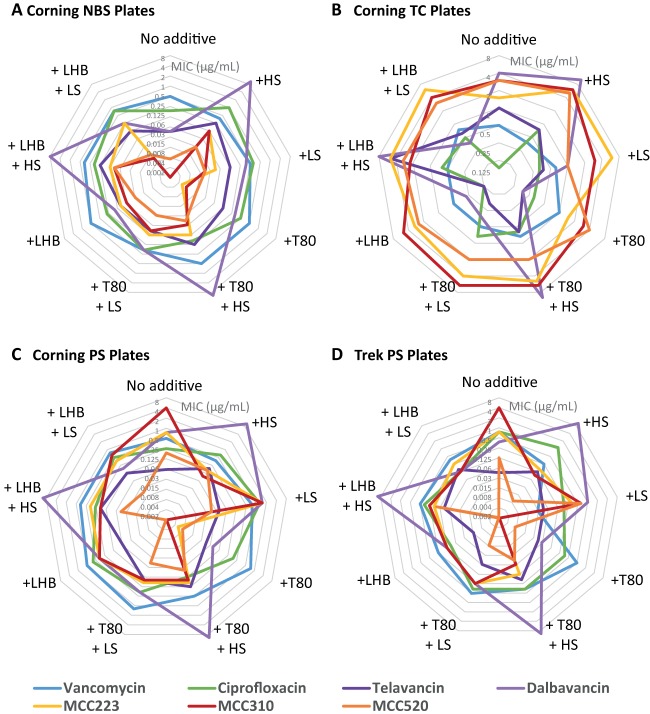
Comparison of antibiotic MICs against MRSA (ATCC 43300) determined in four plate types with and without the addition of 0.002% Tween 80 or LHB, with and without the addition of human serum or lung surfactant, as follows: Corning NBS (A), Corning TC (B), Corning PS (C), and Trek PS (D). Note that each hexagon represents one antibiotic-strain pair. If the MIC remains constant across plate types/conditions, the plot should be symmetrical, as is generally the case for vancomycin (blue).

The Gram-positive antibiotic panel against MRSA ATCC 43300 (Table 4 and Fig. 6) demonstrated again that vancomycin and ciprofloxacin had little variation with plate type or any combination of T80, LHB, HS, or LS additive. Dalbavancin was strikingly inactivated in the presence of 50% HS under all plate type and additive conditions, with telavancin and vancapticin activities reduced to a lesser extent. In TC plates, T80 and LHB improved the activities of telavancin and dalbavancin but had little effect on vancapticin activity. In PS plates, telavancin was generally unaffected by the additives, while dalbavancin and the vancapticins showed significant improvement (>10-fold). NBS plates gave the most potent activity for all lipoglycopeptides but with reductions in activity when T80 or LHB was added.

**TABLE 4:** Comparison of 4 plate types in Mueller-Hinton broth versus MRSA ATCC 43300 in the presence and absence of Tween 80 (polysorbate 80) or lysed horse blood (LHB), with added 50% human serum (HS) or 2% lung surfactant (LS)

Plate	Medium	Additive	MIC (µg/ml) by antibiotic						
			Vancomycin	Ciprofloxacin	Telavancin	Dalbavancin	MCC223	MCC310	MCC520
Corning NBS	MH	None	0.5	0.125 to 0.25	0.03 to 0.06	0.03 to 0.06	≤0.003	≤0.003	0.007
		50% HS	0.25 to 0.5	1	0.25	>8	0.125	0.125	0.03
		2% LS	0.5	0.125 to 1	0.125	0.25 to 0.5	0.03 to 0.06	0.015 to 0.03	0.015 to 0.03
	MH + T80	None	1	0.5	0.125	0.125 to 0.5	0.003 to 0.007	0.007	0.015
		50% HS	1	0.125 to 0.25	0.25	>8	0.125	0.06	0.03 to 0.06
		2% LS	0.25 to 0.5	0.25 to 0.5	0.06 to 0.125	0.25 to 0.5	0.125	0.06 to 0.125	0.03
	MH + 2% LHB	None	1	0.06 to 0.5	0.06 to 0.125	0.06 to 0.25	0.06 to 0.125	0.06	0.03
		50% HS	0.5 to 1	0.25 to 0.5	0.25	8	0.125	0.06 to 0.125	0.06 to 0.125
		2% LS	0.5 to 1	0.5 to 1	0.125	0.25	0.25	0.015 to 0.06	0.015
Corning PS	MH	None	0.5	0.25	0.06	0.5 to 1	0.5 to 1	4	0.125 to 0.25
		50% HS	0.25 to 0.5	0.25 to 1	0.125 to 0.25	>8	0.06 to 0.25	0.06 to 0.125	0.06 to 0.25
		2% LS	0.5 to 1	0.5 to 2	0.03 to 0.125	1 to 2	1 to 2	1 to 2	0.03 to 0.06
	MH + T80	None	1 to 2	0.25 to 0.5	0.03 to 0.06	0.03 to 0.125	0.003 to 0.007	≤0.003	0.007
		50% HS	0.5	0.125	0.25	>8	0.125 to 0.25	0.06 to 0.125	0.03 to 0.125
		2% LS	0.5 to 2	0.25 to 0.5	0.06 to 0.25	0.25 to 0.5	0.125 to 0.25	0.06 to 0.25	0.03 to 0.06
	MH + 2% LHB	None	1	0.25 to 1	0.06	0.25 to 0.5	0.25 to 0.5	0.25 to 0.5	≤0.003
		50% HS	0.5 to 1	0.03 to 0.5	0.125 to 0.25	>8	0.25 to 0.5	0.125 to 0.25	0.03 to 0.06
		2% LS	0.5 to 1	0.5	0.125	0.125 to 0.25	0.25 to 0.5	0.25 to 1	0.015 to 0.03
Corning TC	MH	None	0.5	0.06 to 0.125	1	4	1 to 2	2 to 4	2 to 4
		50% HS	0.25 to 0.5	0.5 to 1	0.5 to 1	>8	4 to >8	4 to >8	1 to >8
		2% LS	0.25 to 1	0.125 to 0.5	0.25 to 0.5	1	4 to 8	2 to 4	1
	MH + T80	None	1	0.125 to 0.5	0.125 to 0.25	0.125 to 0.25	1 to 2	2 to 4	4
		50% HS	0.5 to 1	0.25 to 1	0.25 to 1	>8	2 to 8	4 to 8	2
		2% LS	0.5	0.5 to 1	0.125 to 0.25	0.25 to 0.5	4	4 to 8	2
	MH + 2% LHB	None	0.5	0.125	0.125	0.06 to 0.5	2 to 4	2 to 8	1 to 4
		50% HS	0.5	0.25 to 1	2 to 8	8	2 to 8	0.5 to 4	2 to 4
		2% LS	0.5 to 1	0.5	0.25 to 1	0.25 to 0.5	4 to >8	4	2 to 4
Trek PS	MH	None	0.5 to 1	0.5 to 1	0.03 to 0.06	0.25 to 0.5	0.5 to 1	4	0.125
		50% HS	0.25	0.25 to 2	0.125	>8	0.06 to 0.25	0.06 to 0.125	0.003 to 0.015
		2% LS	0.03	0.125 to 0.25	0.03 to 0.06	1	0.25 to 0.5	0.25 to 1	0.25 to 1
	MH + T80	None	1	0.25 to 0.5	0.03 to 0.06	0.06	≤0.003	≤0.003	0.007
		50% HS	0.25 to 0.5	0.25 to 0.5	0.125 to 0.25	8 to >8	0.125	0.06	0.03 to 0.06
		2% LS	0.06 to 1	0.25 to 0.5	0.06	0.25	0.25	0.25	0.015
	MH + 2% LHB	None	0.25	0.06 to 0.125	0.015	0.125	0.125	0.125	0.003 to 0.015
		50% HS	0.5	0.25 to 0.5	0.06 to 0.125	8 to >8	0.125 to 0.25	0.06 to 0.5	0.06 to 0.25
		2% LS	0.25 to 0.5	0.125	0.06 to 0.25	0.125	0.25	0.125	≤0.003

**FIG 7:**
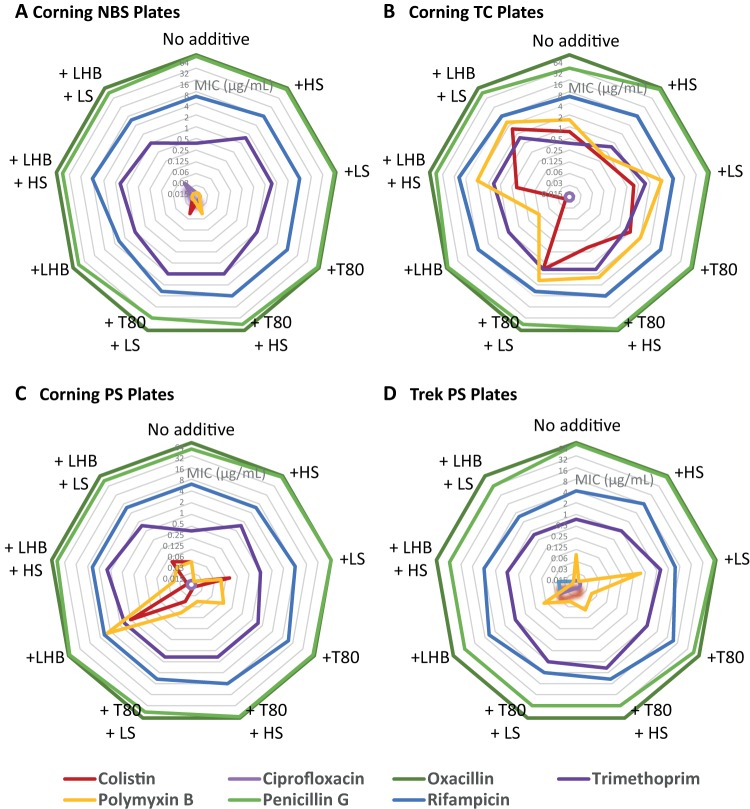
Comparison of antibiotic MICs against *E. coli* (ATCC 25922) determined in four plate types with and without the addition of 0.002% Tween 80 or LHB, with and without the addition of human serum or lung surfactant, as follows: Corning NBS (A), Corning TC (B), Corning PS (C), and Trek PS (D). Note that each hexagon represents one antibiotic-strain pair. If the MIC remains constant across plate types/conditions, the plot should be symmetrical, as is generally the case for rifampin (blue).

In the Gram-negative antibiotic panel (Table 5 and Fig. 7), ciprofloxacin, oxacillin, penicillin G, trimethoprim, and rifampin again showed little variation even with HS or LS additives. Trimethoprim was marginally more active in their absence across multiple plate types, though this difference disappeared once T80 or LHB was added. Colistin and polymyxin B were most active in NBS plates and least active in TC plates regardless of T80, LHB, HS, or LS additive. Curiously, LHB in Corning PS plates greatly reduced colistin and polymyxin B activity compared to T80 but not in Trek PS plates, and in TC plates, the effect was reversed.

**TABLE 5:** Comparison of 4 plate types in Mueller-Hinton broth versus *E. coli* ATCC 25922 in the presence and absence of Tween 80 (polysorbate 80) or lysed horse blood (LHB), with added 50% human serum (HS) or 2% lung surfactant (LS)

Plate	Medium	Additive	MIC (µg/ml) by antibiotic						
			Colistin	Ciprofloxacin	Oxacillin	Trimethoprim	Polymyxin B	Penicillin G	Rifampin
Corning NBS	MH	None	≤0.03	≤0.03	>64	0.25 to 0.5	≤0.03	64	4 to 8
		50% HS	≤0.03	≤0.03	>64	1 to 2	≤0.03	64	8
		2% LS	≤0.03	≤0.03	>64	1 to 2	≤0.03	64	8
	MH + T80	None	≤0.03	≤0.03	>64	1	≤0.03	64	8
		50% HS	≤0.03	≤0.03	>64	2	0.03 to 0.06	32 to 64	8
		2% LS	0.03 to 0.06	≤0.03	>64	2	≤0.03	32	4 to 8
	MH + 2% LHB	None	≤0.03	≤0.03	>64	1	≤0.03	32 to 64	2 to 4
		50% HS	≤0.03	≤0.03	>64	1 to 2	≤0.03	32 to 64	8
		2% LS	0.03 to 0.06	0.03 to 0.06	>64	1	≤0.03	32 to 64	4 to 8
Corning PS	MH	None	0.06	≤0.03	>64	0.25 to 0.5	0.06	32 to 64	4 to 8
		50% HS	≤0.03	≤0.03	>64	1 to 2	≤0.03	64	4 to 8
		2% LS	0.06 to 0.25	≤0.03	>64	1	0.06 to 0.125	64 to >64	8
	MH + T80	None	≤0.03	≤0.03	>64	1 to 2	0.03 to 0.25	64	8 to 16
		50% HS	≤0.03	≤0.03	>64	1 to 2	0.03 to 0.06	64	8
		2% LS	0.03 to 0.06	≤0.03	>64	1 to 2	0.06 to 0.125	32 to 64	4 to 8
	MH + 2% LHB	None	1	≤0.03	>64	1 to 2	2 to 8	64	4 to 8
		50% HS	≤0.03	≤0.03	>64	1 to 8	0.03 to 0.06	32 to >64	4 to 8
		2% LS	0.06 to 0.125	≤0.03	>64	1 to 2	0.06	32 to 64	4 to 8
Corning TC	MH	None	0.5 to 1	≤0.03	>64	0.25 to 0.5	1 to 2	32	4 to 8
		50% HS	0.125 to 0.5	≤0.03	>64	0.5 to 1	0.25 to 0.5	64	8
		2% LS	0.5 to 1	≤0.03	>64	1 to 2	4	64	8
	MH + T80	None	1	≤0.03	>64	0.5 to 1	2	64	8
		50% HS	0.25 to 0.5	≤0.03	>64	1 to 2	1 to 4	64	8
		2% LS	1 to 2	≤0.03	>64	1 to 2	2 to 4	32 to 64	4 to 8
	MH + 2% LHB	None	≤0.03	≤0.03	>64	1	0.125	64	8
		50% HS	0.25 to 0.5	≤0.03	>64	1 to 2	4	32 to 64	8 to 16
		2% LS	2 to 4	≤0.03	>64	1 to 2	2 to 8	32 to 64	8
Trek PS	MH	None	≤0.03	≤0.03	>64	0.5 to 1	0.06 to 0.125	64	4
		50% HS	≤0.03	≤0.03	>64	1	≤0.03	64 to >64	8
		2% LS	≤0.03	≤0.03	>64	1 to 4	0.5 to 1	64	4 to 8
	MH + T80	None	≤0.03	≤0.03	>64	2	0.03 to 0.06	32 to 64	8 to 16
		50% HS	≤0.03	≤0.03	>64	2 to 4	0.03 to 0.125	32	4 to 8
		2% LS	≤0.03	≤0.03	>64	2	0.03 to 0.06	32	4
	MH + 2% LHB	None	0.03 to 0.06	0.03 to 0.06	>64	1	0.03 to 0.25	32	4 to 8
		50% HS	0.03 to 0.06	≤0.03	>64	1	≤0.03	32	4
		2% LS	≤0.03	≤0.03	>64	0.5 to 1	≤0.03	32	2 to 4

## DISCUSSION

We have, for the first time, systematically evaluated plate-based effects on MIC determinations during broth microdilution assays. While MIC assay guidelines cover a range of experimental parameters, the composition of the assay vessel is generally not specified, other than the CLSI-EUCAST recommendations for colistin that state that plain PS should be employed (7).

The majority of antibiotic stock solutions for testing are prepared in water, phosphate buffer, or pH-adjusted aqueous solution (106 out of 139 reported in CLSI M100 [see Table 6A, “Solvents and diluents for preparation of stock solutions of antimicrobial agents”]) (3). However, several antibiotics, notably the lipophilic lipoglycopeptides telavancin (compound 2), dalbavancin (compound 3), and oritavancin (compound 4) (Fig. 1), must be solubilized in DMSO or 0.002% polysorbate 80 (Tween 80) in water (3). Furthermore, the CLSI reference MIC quality control range tabulated for dalbavancin and oritavancin are obtained in cation-adjusted Mueller-Hinton broth (CAMHB) supplemented with 0.002% polysorbate 80 (see Table 5A-1 in CLSI M100, “MIC QC ranges for nonfastidious organisms and antimcirobial agents”) (3). This requirement is consistent with published reports that 0.002% (final concentration) of polysorbate 80 is required for reproducible MIC testing of dalbavancin without substrate or medium constituent interference, due to poor antibiotic solubility and facile absorption to plastic surfaces (13). If the dalbavancin dilutions were in contact with plastic for as little as 30 min before inoculation with surfactant-containing media, the measured MIC against S. aureus ATCC 29123 rose from CLSI-consistent values of 0.06 µg/ml to values of 2 to 8 µg/ml. Similarly, oritavancin (compound 4) MIC values were underestimated by 16- to 32-fold in the absence of added polysorbate 80, again due to the depletion of free drug onto plastic surfaces (10, 11). The loss to PS microtiter plates was quantified using [^14^C]oritavancin; at 16 µg/ml, the concentration of oritavancin was approximately 70% of that expected, but at 4 µg/ml, it was only 35%, and at 1 µg/ml, it was <10% (10). The addition of 2% lysed horse blood (LHB) was found to have the same blocking effect as surfactant (10). A similar effect, with added protein reducing antibiotic adherence to plastic, was observed with the glycolipodepsipeptide complex ramoplanin (compound 6), where the addition of 0.02% bovine serum albumin (BSA) resulted in more potent MICs (15, 16). In 2014, the CLSI methods for determining the MIC of telavancin (compound 2) were revised to include the addition of polysorbate 80, with DMSO used during stock preparation (12, 14). Notably, the closely related glycopeptide vancomycin (compound 5), without a lipophilic moiety, does not require surfactant supplement. A recent report discussed the effects of solvent (DMSO, ethanol, and methanol) on bacterial growth and found 20% reductions in growth across five organisms at concentrations of >3% DMSO, >3% methanol, or 1% ethanol, so solubilizing additives may also affect assay results (26).

Members of the lipopeptide polymyxin class of antibiotics (polymyxin B [compound 7a] and polymyxin E [or colistin, compound 7b]) are important last-line therapeutic agents against many multidrug-resistant Gram-negative bacteria (27–31). They consist of an *N*-terminal fatty acid side chain that is attached to a polycationic *deca*-peptide backbone (Fig. 1) with physicochemical properties similar to those of the lipoglycopeptides. These structural features confer amphipathicity, which is a key feature of many other cationic antimicrobial peptides (CAMPs) (32–34). As mentioned earlier, plate types have been reported to affect CAMP MIC values (8). In 2012, the addition of 0.002% polysorbate was reported to improve the MIC results for colistin and polymyxin B, with 4- to 8-fold more potent MICs against over 200 strains with surfactant present (19), with the results confirmed in clinical isolates (17, 18, 20). Greater differences were observed when the initial MIC was lower (<2 µg/ml). As for oritavancin, measurement of colistin concentrations in MHB following incubation in PS, PP, and glass tubes showed substantial time-dependent depletion at lower concentrations, with only 8%, 23%, or 25% of the initial 0.125 µg/ml concentration detected after 24 h, but 84%, 90%, and 80% of an expected 8 µg/ml concentration detected, respectively (21). Dramatic differences were observed between different brands of untreated PS microwell plates, comparing those from Greiner (remarkably, only 2% of 8 µg/ml after 24 h) and Nunc (70% of 8 µg/ml after 24 h). Low-binding PP microtubes showed the least loss at low concentrations (59% of 0.125 µg/ml after 24 h) (21). However, a CLSI-EUCAST working group in 2016 determined that surfactants should not be included in the reference broth microdilution method for colistin, and that untreated PS trays should be employed (7). Two new reports in 2018 described container effects on polymyxin activity. Untreated PS Sensititre GNX2F assay plates (Thermo Fisher) were compared to broth macrodilution in borosilicate glass against 106 carbapenem-resistant strains of Klebsiella pneumoniae, with 97% agreement within one dilution for polymyxin B and 92% for colistin (23). PS and glass-coated plates were compared for broth microdilution assays of colistin and polymyxin against 42 carbapenem-resistant strains of Acinetobacter baumannii (22). For both antibiotics, the PS resulted in greater variability and slightly less potent MICs (glass plate MIC for all 42 strains, 1 or 2 µg/ml; PS plates had 3 isolates with MIC of 2 µg/ml).

Other assay additives can affect MIC values. The importance of plasma protein binding on drug effectiveness is contentious: while it is generally recognized that the free drug concentration correlates with on-target activity, the reduced concentration of free drug caused by higher protein binding is offset by increased overall exposure due to protection from hepatic and nonhepatic clearance (35, 36). Plasma protein binding has been implicated as a major factor limiting the active free concentration of many clinically important antibiotics (37–42). This in turn translates into reduced antibacterial activity and clinical dose escalation that, in certain cases where the antibacterial agent is highly bound, limits its intravenous use (40, 43, 44). Most antibiotics generally have protein binding values of <60% (e.g., aminoglycosides, 0 to 35% for four examples [45]; the carbapenem meropenem, 2% [46]; the oxazolidinone linezolid, 31% [47]; tetracyclines, 24 to 60% for four examples [48]; fluoroquinolones in general, ∼30% [49]; and the glycopeptide vancomycin, 55% [50]). However, the second-generation lipoglycopeptide antibiotics dalbavancin and oritavancin, both approved in 2014, have high human protein binding, estimated at 93 (51) to >95% (52) and 82 to 90% (50, 53), respectively, which leads to exceedingly long half-lives (estimated at 9 to 12 [51, 54] and 10 to 17 [50] days) with once-weekly or even single-injection dosing. Telavancin also has high binding (90 to 93% [55, 61]), though a much shorter half-life (8 h [50]), requiring once-daily dosing. In contrast, the polymyxin lipopeptides colistin and polymyxin B are estimated to have 50 to 60% plasma protein binding (56, 57). Daptomycin is another cationic lipopeptide with high protein binding (92% [58]).

The extent of protein binding of antibiotics is often approximated by a serum reversal MIC instead of standard equilibrium dialysis or ultrafiltration methods. This technique conducts MIC assays without and with added serum proteins, either with broth containing 50 to 95% human or mouse serum, or with added 3 to 4% human serum albumin or bovine serum albumin protein (53, 59, 60). The ratio of retained activity indicates the extent of unbound antibiotic. However, bacteria do not grow as well in human serum as in standard medium, so high concentrations of serum may have a synergistic antimicrobial effect (59). MIC assays of lipoglycopeptides/lipopeptides that bind to both protein and plastic have the potential to be confounded by the opposing effects. High protein binding means that little free antibiotic is available for antimicrobial activity, reducing their MIC potency, but the added protein also reduces nonspecific binding to plastic, resulting in more potent MIC values.

We now demonstrate that plate type can cause large variations in MIC assay results, not only between different types of plate composition/coatings but sometimes in plates of the same polymer from different manufacturers. This supports the previously reported dramatic variation in colistin concentrations when incubated in untreated PS plates from different manufacturers (21). It is evident that to enable an “apples-to-apples” comparison of data from different laboratories, the exact plate type and composition (or vessels used for macrodilution experiments) should be reported when describing MIC assays.

The extent of plate-based variations is highly dependent on the type of antibiotic and appears to correlate with hydrophobic or amphiphilic molecules. We have found that for antibiotics where the addition of Tween 80 leads to more potent activity, the use of NBS plates without additive provides similar results. This suggests that the reduced activity in PS plates is caused by loss of compound due to binding to the plate surface, with even greater loss of compound in TC plates. However, there is a disconnect between the extent of protein binding of antibiotics and their “stickiness” to plastic, based on the observed plate effects. Telavancin (90 to 93% protein binding) showed little alterations in MIC when tested in NBS versus untreated plates, while dalbavancin (93 to >95%) and colistin (60%) both resulted in large variations.

In summary, plate and additive effects are observed across a range of bacteria, but there are subtle variations depending on the antibiotic, plate type, and additives employed. All broth microdilution MIC determinations should clearly specific the plate type and manufacturer and any additives employed. These studies demonstrate that NBS plates can effectively prevent reductions in MIC due to adsorption of compound to the plate surface and allow for assays of lipophilic antibiotics without the need for added surfactant. We are conducting further investigations against a much larger panel of antibiotics to establish the extent of plate-based variations in MIC determinations. It remains to be determined which plate type provides the “true” MIC value that is most relevant to the clinical activity of the antibiotic.

## MATERIALS AND METHODS

### Materials.

Vancomycin (catalog no. 861987-250MG, lot 087K0694), ciprofloxacin (catalog no. 17850-25G-F, lot 0001396108), oxacillin (catalog no. O1002-1G, lot 018K0610), penicillin G (catalog no. 13752-1G-F, lot WE376306/1), trimethoprim (catalog no. T7883-5G, lot 078K1522), colistin (catalog no. C4461-1G, lot 036K1374, 15,000 units per mg), polymyxin B (catalog no. P0972-10MU, lot 453306, ≥6,000 USP units per mg), rifampin (catalog no. R3501-250MG), polysorbate 80 (Tween 80, catalog no. P8074-500ml), and human serum (catalog no. H4522-20ml) were obtained from Sigma-Aldrich (Sydney, NSW, Australia). Dalbavancin HCl (catalog no. 317136) was purchased from MedKoo Bioscience, MHB (catalog no. 211443) from Bacto Laboratories, beractant (Survanta) from Abbvie Pty Ltd. (catalog no. 1039.008), and lysed horse blood (catalog no. HB100) from Thermo Fisher Scientific (Australia).

Telavancin (61) was synthesised from vancomycin according to procedures in the literature. MCC223, MCC310, and MCC520 were synthesized from vancomycin and purified by high-performance liquid chromatography (HPLC) to >95% purity (24). Their purity was ascertained by analytical liquid chromatography-mass spectrometry (LC-MS) and identity confirmed by high-resolution MS (HRMS) and tandem MS (MS/MS) fragmentation.

The Gram-positive bacteria Staphylococcus aureus (methicillin-sensitive S. aureus [MSSA] ATCC 25923, MRSA ATCC 43300, vancomycin-intermediate S. aureus [VISA] NRS 2/ATCC 700698), Streptococcus pneumoniae (ATCC 33400, multidrug-resistant [MDR] ATCC 700677), Enterococcus faecalis (ATCC 29212) and Streptococcus pyogenes (ATCC 12344), and Gram-negative bacteria Escherichia coli (ATCC 25922), Klebsiella pneumoniae (ATCC 13883), Pseudomonas aeruginosa (ATCC 10145), and Acinetobacter baumannii (ATCC 19606) were sourced from the American Type Culture Collection (ATCC) and Network on Antimicrobial Resistance in Staphylococcus aureus (NARSA).

The plate types used were 96-well PS Corning flat-bottom untreated Costar 3370, Corning flat-bottom TC surface COR 3628, Corning flat-bottom NBS surface COR 3641, Thermo Electric flat-bottom untreated PS Nunc-442404, Thermo Electric flat-bottom TC surface Nunc-167008, Thermo Electric U-bottom TC surface Nunc-163320, and Trek Diagnostics untreated PC H511A.

### MIC determination via broth microdilution assay.

MIC determinations were done in duplicate (*n* = 2), with vancomycin, telavancin, dalbavancin, and ciprofloxacin used as positive inhibitor controls for Gram-positive bacteria, and colistin, ciprofloxacin, oxacillin, trimethoprim, polymyxin B, penicillin G, and rifampin for Gram-negative bacteria. A positive control of a row of just the bacteria and a negative control of only the medium were included for every plate tested. The antibiotic standards were prepared to 1.28 mg/ml solution in water. MCC223, MCC310, and MCC520 were prepared to 160 µg/ml solution in water from a stock solution of 1 mM concentration.

The compounds along with standard antibiotics were serially diluted 2-fold across the 96-well plates. Standards ranged from 64 µg/ml to 0.03 µg/ml and compounds from 8 µg/ml to 0.003 µg/ml, with final volumes of 50 µl per well. Gram-positive and Gram-negative bacteria were cultured in Mueller-Hinton broth (catalog no. 211443; Bacto Laboratories) with and without 0.002% Tween at 37°C overnight. A sample of each culture was then diluted 40-fold in fresh MH broth (in presence and absence of Tween) and incubated at 37°C for 2 to 3 h. The resultant mid-log-phase cultures were diluted to 1 × 10^6^ CFU/ml under the same two conditions, and then 50 µl was added to each well of the compound-containing 96-well plates to give a final cell density of 5 × 10^5^ CFU/ml. All the plates were covered and incubated at 37°C for 24 h. MICs were determined visually at 24 h of incubation, with the MIC defined as the lowest concentration at which no growth was visible after incubation.

For experiments in the presence of surfactant, 2% beractant (25 mg/ml) was added to the mid-log-phase cultures and mixed gently and added to all the 96-well plates. For experiments in the presence of serum, a mixture of 50% of human serum along with 50% MHB was prepared and used throughout the experiment.

## Supplementary Material

Supplemental file 1
