# Supplementary material for: Effects of Microplate Type and Broth Additives on Microdilution MIC Susceptibility Assays
Source: Antimicrob Agents Chemother. 2018 Dec 21;63(1):e01760-18. doi: 10.1128/AAC.01760-18 (PMC6325200; doi:10.1128/AAC.01760-18)
Supplement: Supplemental file 1 [file 50af9bb6786b4530120bc558db62b77c_AAC.01760-18-s0001.pdf]

# Supplemental Information

## Effects of Microplate Type and Broth Additives on Microdilution MIC Susceptibility Assays

Angela Kavanagh,* Soumya Ramu,* Yujing Gong, Matthew A Cooper# and Mark A. T. Blaskovich#

Institute for Molecular Bioscience, The University of Queensland, St Lucia, Queensland, Australia.

Running Head: Plate and Additive Effects on Broth MIC Determinations

# Address correspondence to Mark A.T. Blaskovich, m.blaskovich@uq.edu.au, and Matthew A. Cooper, m.cooper@uq.edu.au

* A.K., and S.R. contributed equally to this work.

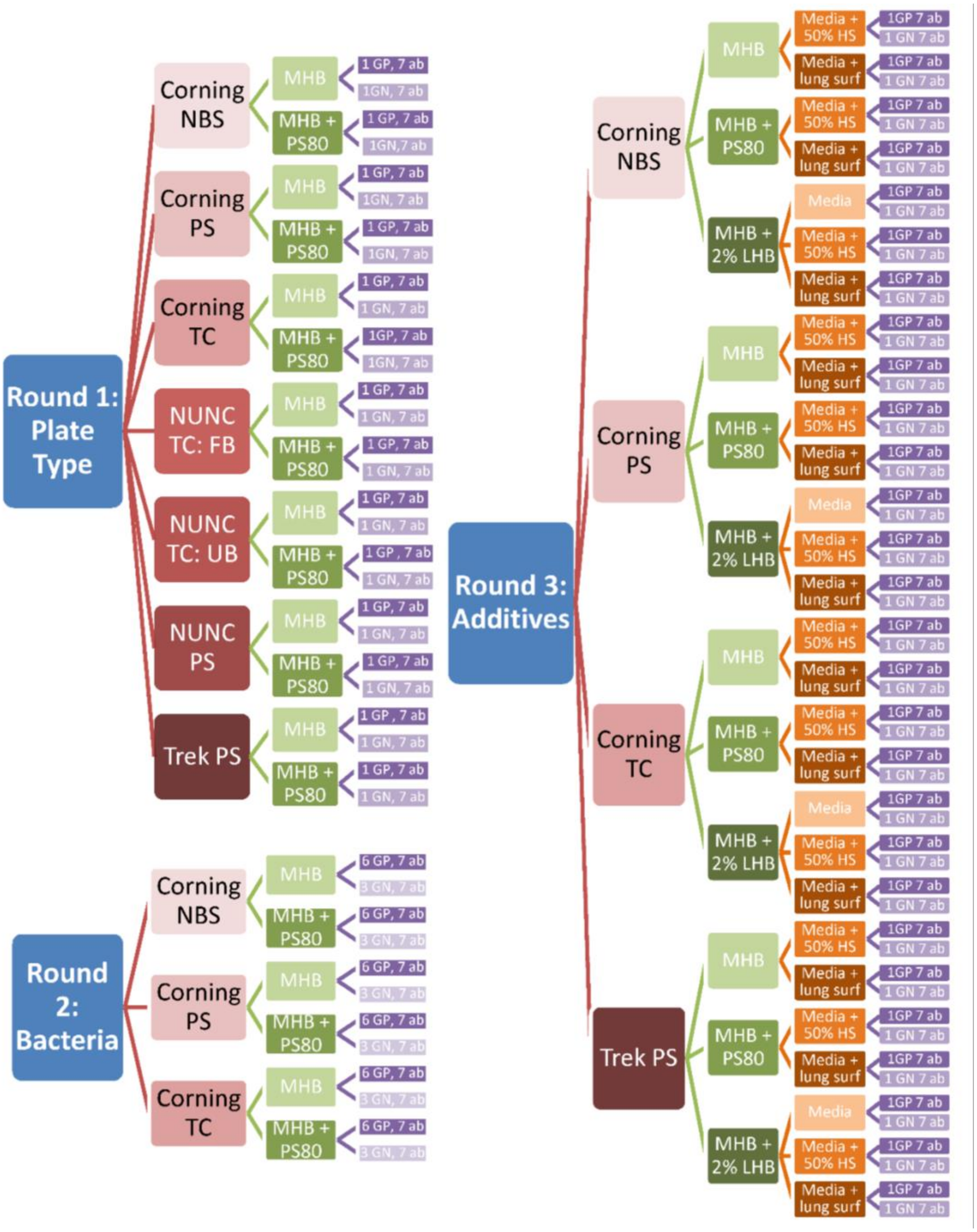


**Figure S1.** Experimental Design

(Ab = antibiotic, GP = Gram-positive bacteria, GN = Gram-negative bacteria, HS = human serum, LHB = lysed horse blood, MHB = Mueller-Hinton Broth, PS = polysorbate 80)

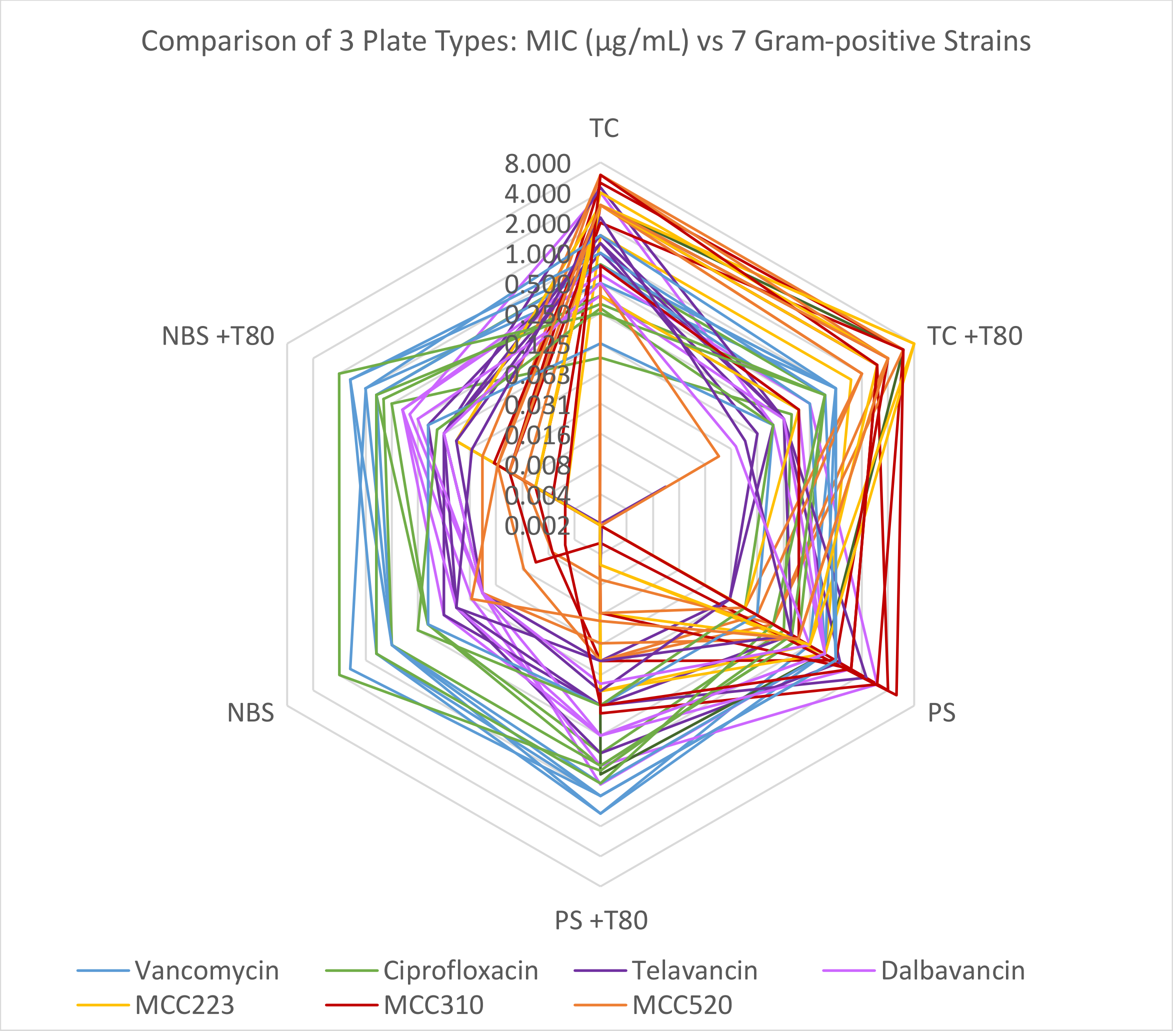


**Figure S2.** Comparison of antibiotic MICs determined in three plate types with and without the addition of 0.002% Tween-80, against 7 strains of Gram-positive bacteria (MRSA, MSSA, GISA, *S. pneumoniae*, MDR *S. pneumoniae, S. pyogenes, E. faecalis*). Note: each hexagon represents one antibiotic-strain pair, with the same colour used for one antibiotic against all 7 strains. If the MIC remains constant across plate types/conditions, the plot should be symmetrical, as for vancomycin (blue).

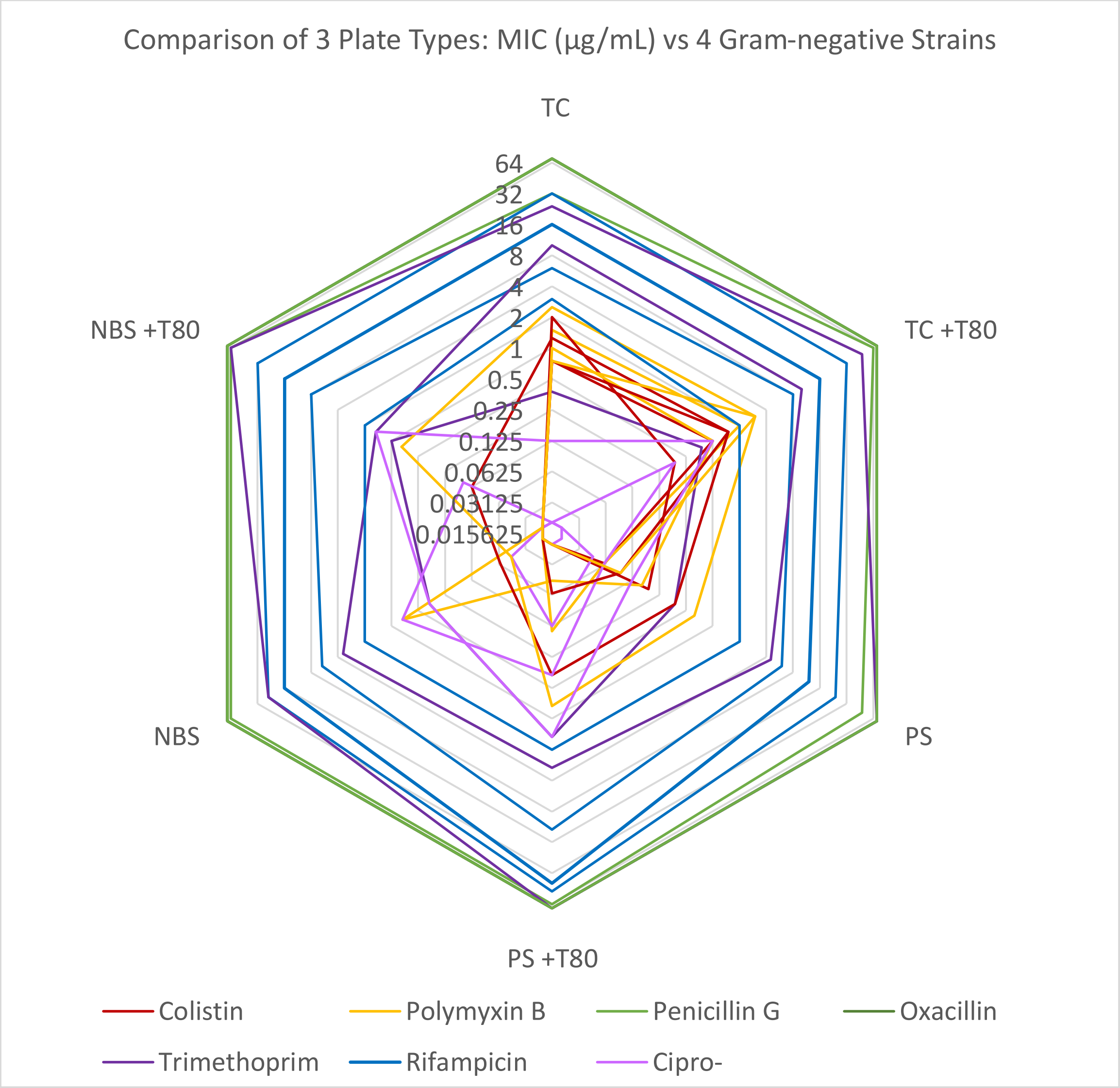


**Figure S3.** Comparison of antibiotic MICs determined in three plate types with and without the addition of 0.002% Tween-80, against 4 strains of Gram-negative bacteria (*E. coli*, *K. pneumoniae*, *A. baumannii*, *P. aeuruginosa*). Note: each hexagon represents one antibiotic-strain pair, with the same colour used for one antibiotic against all 7 strains. If the MIC remains constant across plate types/conditions, the plot should be symmetrical, as for rifampicin (blue).

**Table S1.** Comparison of 3 Plate Types vs Seven Gram Positive Bacteria in Mueller Hinton Broth in the presence and absence of Tween-80 (polysorbate 80) (MIC, µg/mL)

| Bacteria | Plate | Media | Antibiotic | | | | | | |
|---|---|---|---|---|---|---|---|---|---|
| | | | Vancomycin | Ciprofloxacin | Telavancin | Dalbavancin | MCC223 | MCC310 | MCC520 |
| MRSA *S. aureus* ATCC 43300 | Corning | -T80 | 0.5 | 0.125-0.25 | 0.03-0.06 | 0.03-0.06 | ≤0.003 | ≤0.003 | 0.007 |
| | NBS | +T80 | 1 | 0.5 | 0.125 | 0.125-0.5 | 0.003-0.007 | 0.007 | 0.015 |
| | Corning | -T80 | 0.5 | 0.06-0.125 | 1 | 4 | 1-2 | 2-4 | 2-4 |
| | TC | +T80 | 1 | 0.125-0.5 | 0.125-0.25 | 0.125-0.25 | 1-2 | 2-4 | 4 |
| | Corning | -T80 | 0.5 | 0.25 | 0.06 | 0.5-1 | 0.5-1 | 4 | 0.125-0.25 |
| | PS | +T80 | 1-2 | 0.25-0.5 | 0.03-0.06 | 0.03-0.125 | 0.003-0.007 | ≤0.003 | 0.007 |
| MSSA *S. aureus* ATCC 25923 | Corning | -T80 | 0.5-1 | 0.125-0.25 | 0.06-0.125 | 0.06 | ≤0.003 | ≤0.003 | ≤0.003 |
| | NBS | +T80 | 1-2 | 0.5-1 | 0.125-0.25 | 0.25-0.5 | 0.06-0.125 | 0.015-0.03 | 0.03 |
| | Corning | -T80 | 0.5-1 | 0.03-0.125 | 0.5-2 | 0.25-1 | 2-4 | 2-8 | 4-8 |
| | TC | +T80 | 1 | 0.5-1 | 0.25 | 0.5 | 4-8 | 4-8 | 4-8 |
| | Corning | -T80 | 0.5-1 | 0.25-0.5 | 0.25-2 | 2-4 | 0.5-1 | 2-4 | 0.125-0.25 |
| | PS | +T80 | 1-2 | 0.5 | 0.25-0.5 | 0.5 | 0.25-1 | 0.06-0.25 | 0.03-0.06 |
| *S. pneumoniae* ATCC 33440 | Corning | -T80 | 0.5-1 | 0.5 | 0.125 | 0.06-0.125 | ≤0.003 | 0.007-0.015 | 0.03-0.06 |
| | NBS | +T80 | 0.5-1 | 0.25-1 | 0.125 | 0.25-0.5 | 0.06-0.125 | 0.015-0.03 | 0.03-0.06 |
| | Corning | -T80 | 0.5-1 | 0.25-0.5 | 1-2 | 0.5-1 | 2-4 | 2 | 4-8 |
| | TC | +T80 | 1 | 0.25-0.5 | 0.25 | 0.25-0.5 | 8 | 4-8 | 4 |
| | Corning | -T80 | 1 | 0.25-0.5 | 0.5-4 | 1-2 | 0.5-1 | 2-8 | 0.125-0.25 |
| | PS | +T80 | 1 | 0.5 | 0.125 | 0.25 | 0.06-0.125 | 0.003-0.06 | 0.03 |
| *S. pyogenes* ATCC 12344 | Corning | -T80 | 0.125-0.25 | 0.25 | ≤0.003 | ≤0.003 | ≤0.003 | ≤0.003 | ≤0.003 |
| | NBS | +T80 | 0.125-0.25 | 0.06-0.25 | 0.003-0.015 | ≤0.003 | ≤0.003 | ≤0.003 | ≤0.003 |
| | Corning | -T80 | 0.125 | 0.06-0.5 | ≤0.003 | ≤0.003 | 0.25-0.5 | 0.5-1 | 0.5 |
| | TC | +T80 | 0.125-0.25 | 0.125-0.25 | 0.007-0.015 | ≤0.003 | 0.25-0.5 | 0.25-0.5 | 0.03-0.06 |
| | Corning | -T80 | 0.125 | 0.06-0.125 | ≤0.003 | ≤0.003 | 0.06-0.125 | 0.25-0.5 | ≤0.003 |
| | PS | +T80 | 0.125 | 0.125 | 0003-0.007 | ≤0.003 | ≤0.003 | ≤0.003 | ≤0.003 |
| *S. pneumoniae* ATCC 700677 | Corning | -T80 | 0.5 | 0.5-1 | 0.03-0.06 | 0.03-0.06 | ≤0.003 | ≤0.003 | ≤0.003 |
| | NBS | +T80 | 1-2 | 0.5-1 | 0.06-0.125 | 0.25 | 0.007-0.015 | 0.007-0.06 | 0.015-0.03 |
| | Corning | -T80 | 1 | 0.125-0.5 | 0.5-2 | 0.25-0.5 | 4 | 2-4 | 2-4 |
| | TC | +T80 | 0.5-1 | 0.5-1 | 0.125 | 0.25 | 4 | 2-4 | 2 |
| | Corning | -T80 | 1 | 0.125-0.25 | 0.06 | 0.5-1 | 0.25-0.5 | 1-2 | 0.06-0.125 |
| | PS | +T80 | 1 | 0.5-1 | 0.06-0.125 | 0.25 | 0.015 | 0.015 | 0.015 |
| *E. faecalis* ATCC 29212 | Corning | -T80 | 1-2 | 2 | 0.06-0.125 | 0.03-0.06 | ≤0.003 | 0.003-0.007 | 0.06 |
| | NBS | +T80 | 1 | 2 | 0.06 | 0.125 | 0.007-0.015 | 0.003-0.007 | 0.03 |
| | Corning | -T80 | 1-2 | 0.25 | 0.5-4 | 0.5 | 2-4 | 4-8 | 2-4 |
| | TC | +T80 | 1 | 0.5-1 | 0.06-0.125 | 0.015-0.125 | 2-4 | 2-4 | 4 |
| | Corning | -T80 | 1 | 0.125-0.5 | 0.125-0.5 | 0.5 | 0.5 | 1-2 | 0.25-0.5 |
| | PS | +T80 | 1 | 0.125-1 | 0.03-0.06 | 0.03-0.125 | 0.003-0.007 | 0.125 | 0.007-0.03 |
| *S. aureus* GISA NRS 2; ATCC 700698 | Corning | -T80 | 0.5 | 64 | 0.06-0.125 | 0.125 | ≤0.003 | 0.007 | 0.015 |
| | NBS | +T80 | 0.5-1 | 64 | 0.125 | 0.25-0.5 | 0.06-0.125 | 0.007-0.015 | 0.03 |
| | Corning | -T80 | 0.5-1 | 32-64 | 1-8 | 0.5-1 | 2-4 | 2-4 | 2-4 |
| | TC | +T80 | 0.5 | 64 | 0.25 | 0.25-0.5 | 8 | 4 | 2 |
| | Corning | -T80 | 0.5-1 | 32->64 | 0.125-0.5 | 0.5-1 | 0.5 | 1 | 0.125 |
| | PS | +T80 | 0.5-1 | 64 | 0.125 | 0.25 | 0.06-0.125 | 0.03-0.06 | 0.03-0.06 |

**Table S2.** Comparison of 3 Plate Types vs Four Gram Negative Bacteria in Mueller Hinton Broth in the presence and absence of Tween-80 (polysorbate 80) (MIC, µg/mL)

| **Bacteria** | **Plate** | **Media** | **Antibiotic** | | | | | | |
|---|---|---|---|---|---|---|---|---|---|
| | | | Colistin | Cipro-floxacin | Oxacillin | Trimeth-oprim | Polymyxin B | Penicillin G | Rifampicin |
| *E. coli* ATCC 25922 | Corning NBS | -T80 | ≤0.03 | ≤0.03 | >64 | 0.25-0.5 | ≤0.03 | 64 | 4-8 |
| | | +T80 | ≤0.03 | ≤0.03 | >64 | 1 | ≤0.03 | 64 | 8 |
| | Corning TC | -T80 | 0.5-1 | ≤0.03 | >64 | 0.25-0.5 | 1-2 | 32 | 4-8 |
| | | +T80 | 1 | ≤0.03 | >64 | 0.5-1 | 2 | 64 | 8 |
| | Corning PS | -T80 | 0.06 | ≤0.03 | >64 | 0.25-0.5 | 0.06 | 32-64 | 4-8 |
| | | +T80 | ≤0.03 | ≤0.03 | >64 | 1-2 | 0.03-0.25 | 64 | 8-16 |
| *K. pneum-oniae* ATCC 13883 | Corning NBS | -T80 | ≤0.03 | 0.03-0.06 | >64 | 4-8 | 0.03-0.125 | >64 | 16 |
| | | +T80 | ≤0.03 | ≤0.03 | >64 | 1-2 | ≤0.03 | >64 | 16 |
| | Corning TC | -T80 | 2 | ≤0.03 | >64 | 4-16 | 1 | >64 | 16 |
| | | +T80 | 0.25-0.5 | ≤0.03 | >64 | 4-16 | 1 | >64 | 16 |
| | Corning PS | -T80 | 0.125-0.25 | 0.03-0.06 | >64 | 1-8 | 0.06-0.25 | >64 | 8-16 |
| | | +T80 | ≤0.03 | 0.125 | >64 | 2-4 | 0.03-0.06 | >64 | 16-64 |
| *P. aeru-ginosa* ATCC 10145 | Corning NBS | -T80 | 0.06 | 0.03-0.125 | >64 | >64 | 0.03-0.06 | >64 | 16-32 |
| | | +T80 | 0.125 | 0.06-025 | >64 | >64 | 0.03-0.125 | >64 | 32 |
| | Corning TC | -T80 | 0.5-2 | ≤0.03 | >64 | >64 | 1-4 | >64 | 32 |
| | | +T80 | 1-2 | 0.25-0.5 | >64 | >64 | 2-4 | >64 | 32 |
| | Corning PS | -T80 | 0.25-0.5 | 0.06 | >64 | >64 | 0.25-1 | >64 | 16-32 |
| | | +T80 | 0.25-0.5 | 0.25-0.5 | >64 | >64 | 0.5-1 | >64 | 32-64 |
| *A. baum-annii* ATCC 19606 | Corning NBS | -T80 | ≤0.03 | 0.25-0.5 | >64 | 16-32 | ≤0.03 | >64 | 2 |
| | | +T80 | ≤0.03 | 1-2 | >64 | 64 | ≤0.03 | >64 | 2 |
| | Corning TC | -T80 | 0.5-1 | 0.125 | >64 | 16-32 | 0.5-1 | >64 | 2-4 |
| | | +T80 | 1-2 | 1 | >64 | 32-64 | 2-4 | >64 | 2 |
| | Corning PS | -T80 | 0.06-0.125 | 0.125 | >64 | >64 | 0.06-0.125 | >64 | 2 |
| | | +T80 | 0.06 | 1-2 | >64 | >64 | ≤0.03 | >64 | 2 |
